# Fatal pediatric Stevens–Johnson syndrome/toxic epidermal necrolysis: Three case reports

**DOI:** 10.1097/MD.0000000000019431

**Published:** 2020-03-20

**Authors:** Tingting Shi, Huan Chen, Li Huang, Huifeng Fan, Diyuan Yang, Dongwei Zhang, Gen Lu

**Affiliations:** a Department of Respiratory; b Department of Digestive; c Pediatric Intensive Care Unit, Guangzhou Women and Children's Medical Center, Guangzhou Medical University, Guangzhou, China.

**Keywords:** children, severe complications, Stevens–Johnson syndrome, toxic epidermal necrolysis

## Abstract

**Rationale:**

Stevens–Johnson syndrome and toxic epidermal necrolysis (SJS/TEN) are extremely rare but potentially life-threatening disorders. We presented 3 fatal pediatric SJS/TEN cases.

**Patient concerns:**

Our patients had some severe complications such as septic shock, respiratory failure and obliterans bronchiolitis (BO) etc.

**Diagnosis:**

Three patients diagnosed SJS/TEN with clinical symptoms that were triggered by antibiotics, nonsteroidal anti-inflammatory drugs, previous infection, or neoplasms.

**Interventions:**

All of them accepted mechanical ventilation, intravenous immunoglobulin (IVIG), blood transfusion, glucocorticoid, and multi-anti-infectious therapy.

**Outcomes:**

They all died because of out-of-control severe infections. In Patient 1, he died 6 days after being admitted to the PICU on the 28th day from onset. In Patient 2, he died on the 211th day from the onset of illness during the third time of PICU admission. In Patient 3, she died 12 days after PICU admission on the 87th day from onset.

**Lessons:**

We should be aware that mucosal damage occurs on the skin and within the mucosa of visceral organs, leading to the occurrence of bronchiectasia, BO, enterocolitis, acute renal failure, and severe secondary infections. Establish a clinically predictive score that includes severe infection for pediatric patients to evaluate the risk of mortality in children in order to improve poor outcomes.

## 1 Introduction

Stevens–Johnson syndrome and toxic epidermal necrolysis (SJS/TEN) are considered a delayed-type hypersensitivity reaction to various agents.^[1]^ They differ in the area of the involved skin, and 74% to 94% of SJS/TEN cases are triggered either by previous medication or an infection.^[2,3]^ SJS/TEN are extremely rare but potentially life-threatening disorders that are characterized by sudden onset of high fever, signs of systemic toxicity, and widespread epidermal necrosis of the skin and mucosa.^[2]^ The morbidity associated with SJS/TEN in children is quite low, that is, from 0.4 to 5.3 per million, but the associated mortality is high, ranging from 16.7% to 44%.^[4–7]^ According to previous reports,^[4,8]^ most children with SJS/TEN died because of multisystem involvement such as respiratory failure, renal failure, septicemia, liver damage, and malignancy. Here, we present three fatal pediatric patients with SJS/TEN and severe complications who presented to the Guangzhou Women and Children's Medical Center. All of them had no history of allergic diseases or other previous illnesses.

### 1.1 Consent statement

Written informed consent was obtained from the parents of patient for the publication of this study (Supplemental Digital Content).

## 2 Case reports

### 2.1 Patient 1

**Figure 1:**
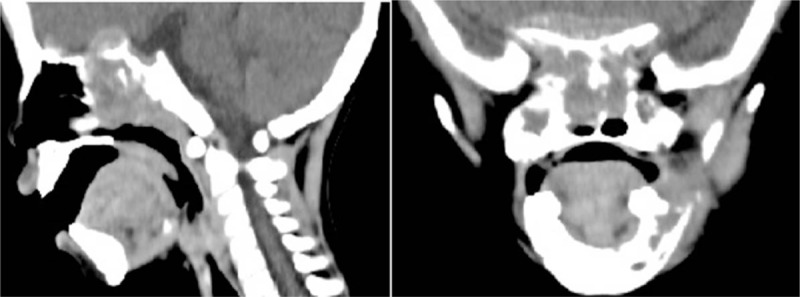
HRCT image showing skull base neoplasms at the nasal cavity and nasopharynx with bone metastasis.

**Figure 2:**
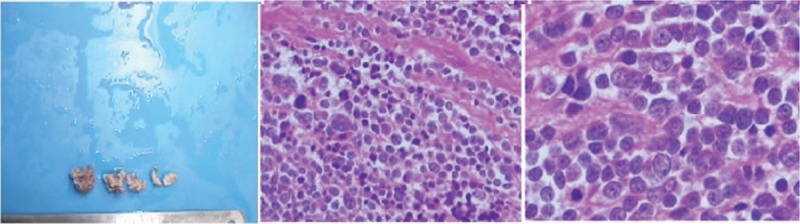
Neoplasm biopsy revealing small round-cell malignant tumors; immunohistochemical study of neuroblastoma (HE staining 200 × , 400 ×) (2B, 2C).

**Figure 3:**
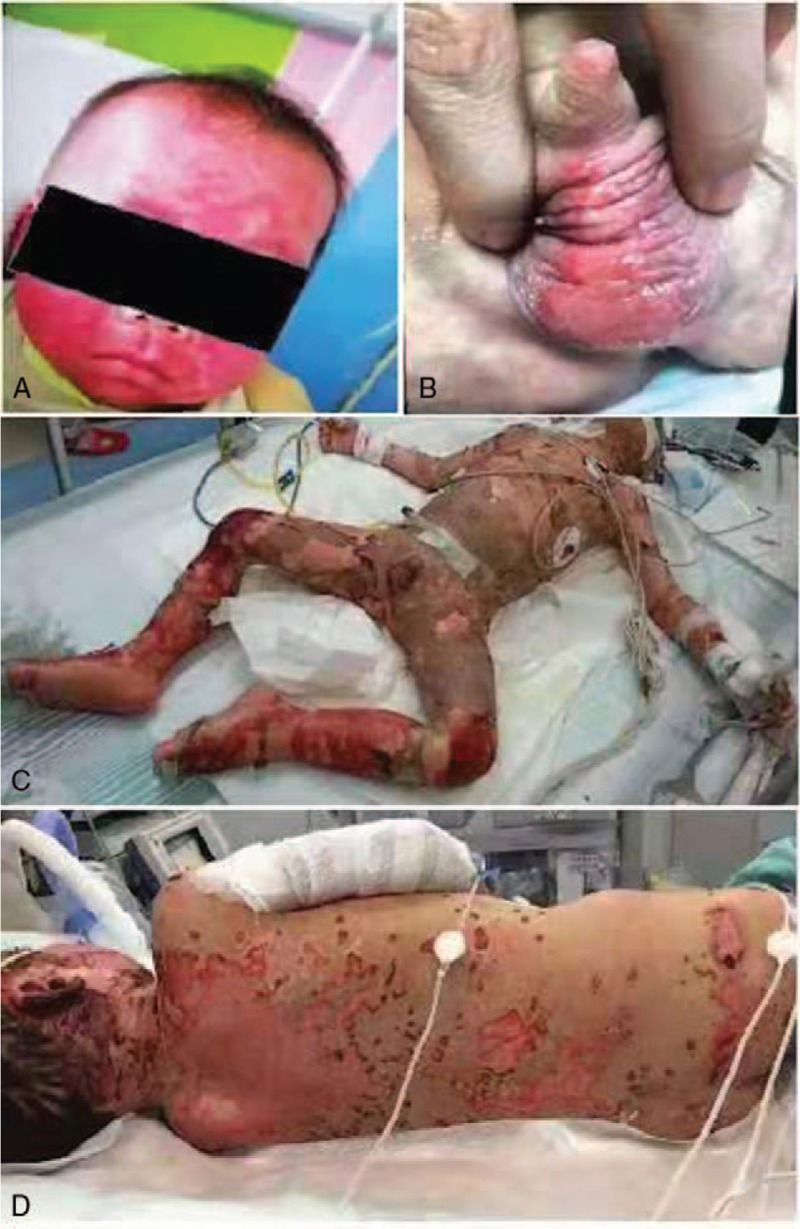
Erythematous papules, bullae, and skin erosions involving the face, neck, genitalia, and trunk, covering approximately 15% BSA (3A, 3B); epidermal detachment involving the face, oral cavity, neck, trunk, and all four limbs, covering approximately 50% BSA (3C, 3D).

A 2-year-old boy was admitted to our hospital because of mild fever and blurred vision for 1 week. He was diagnosed with suspected skull base neoplasms with bone metastasis by high-resolution computed tomography (HRCT) (Fig. 1). To confirm the diagnosis of the neoplasms and control the previous infection, the patient underwent biopsy and recovered antibiotic (cefazolin) treatment. The biopsy of the neoplasms revealed neuroblastoma (small round cell malignant tumors) (Fig. 2). However, his temperature did not improve, and on the 14th day from the onset of illness, erythematous papules, bullae, and skin erosions developed on his face, neck, genitalia, and trunk, covering approximately 15% of his body surface area (BSA) (Fig. 3A, B). Dermatologic consultations were obtained, and the diagnosis of SJS/TEN was considered. He was treated with intravenous immunoglobulin (IVIG) 0.4 g/kg/d and methylprednisolone 10 mg/kg/d for 3 days. However, a larger area of the skin (approximately 25% BSA) became involved within next 6 days. He was transferred to the pediatric intensive care unit (PICU) because of respiratory distress, hematemesis, and severe infection on the 22nd day from onset.

On PICU admission, his axillary temperature was 35.8°C, heart rate was 162 beast/min, respiratory rate was 50 breaths/min, blood pressure was 74/48 mmHg, and oxygen saturation was 92% during high flow oxygen. Physical examination revealed skin lesions covering approximately 25% of his BSA. Chest auscultation was normal. Abdominal distension and hyperactive bowel sounds were detected, and distal extremities were notably cool. Blood test results were as follows: white blood cell (WBC) count, 1.3 × 10^9^/L (NEUT 78%, LYM 19%); platelet count, 28 × 10^9^/L; hemoglobin level, 73 g/L; and C-reactive protein (CRP) level, >200 mg/dL. Serum biochemistry index was normal. Arterial blood gas examination performed on PICU admission revealed pH of 7.27, PaCO_2_ of 37.5 mmHg, and PaO2 of 50 mmHg during high flow oxygen before mechanical ventilation (MV) therapy. Chest and abdominal X-rays were normal.

With the diagnosis of SJS/TEN along with septic shock and respiratory failure, multi-antibiotic (meropenem and vancomycin) and aggressive support treatments were administered. Despite the WBC, platelet, and hemoglobin counts returning to normal, the child rapidly progressively deteriorated with persistent high CRP levels (148–200 mg/dL) and PCT (33.6–77.43 ng/mL). However, no pathogen was detected both in the blood and sputum cultures. He died 6 days after being admitted to the PICU because of out-of-control severe infections on the 28th day from onset.

### 2.2 Patient 2

A 6-year-old boy was prescribed nonsteroidal anti-inflammatory drugs (NSAIDs) by a clinic physician because of mild fever and oral mucosa erosions for 2 days. He developed high-grade fever, bilateral conjunctivitis, and rashes on the 4th day from the onset of illness. He had mild cough and dyspnea on the 6th day from onset. He was brought to the PICU because of respiratory distress and skin lesions with a widespread vesicular rash on the 8th day from onset.

On PICU admission, his axillary temperature was 39.0°C, heart rate was 160 beats/min, respiratory rate was 50 breaths/min, blood pressure was 138/70 mmHg, and oxygen saturation was 85% in room air. Physical examination revealed epidermal detachment involving his face, oral cavity, neck, trunk, and all four limbs covering approximately 50% of his BSA (Fig. 3C, D). Eyelid edema and severe bilateral non-purulent conjunctivitis were also noted. Bilateral crackles were audible in the chest. Blood test results were as follows: WBC count, 24.98 × 10^9^/L (NEUT 84.9%, LYM 10.1%); platelet count, 235 × 10^9^/L; hemoglobin level, 138 g/L; and CRP level, 116.85 mg/dL. Abnormal serum biochemistry index was observed (aspartate aminotransferase [AST], 70 U/L; alanine aminotransferase [ALT], 55 U/L; creatinine, 112 mg/dL). Arterial blood gas examination performed on PICU admission revealed pH of 7.30, PaCO_2_ of 55 mmHg, and PaO_2_ of 90 mmHg during fraction concentration of oxygen in inspired air (FiO_2_) of 0.6 before MV therapy. Chest X-ray revealed bilateral effusion.

**Figure 4:**
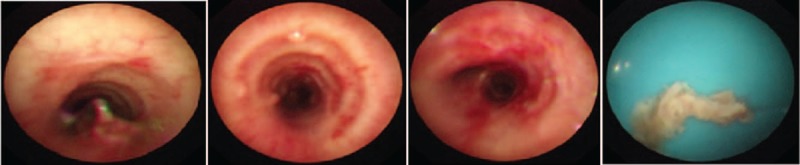
Bronchoscopy image showing bronchial mucosal erosion and inflammatory small nodule formation; floc tissue can be attracted.

Based on the diagnosis of SJS/TEN with respiratory failure, pneumonia, and sepsis, MV therapy and multi-antibiotic (meropenem and vancomycin) therapy were administered. He was treated with IVIG 1 g/kg/d and methylprednisolone 10 mg/kg/d for 2 days with subsequent slow tapering. The child's condition gradually improved. However, on the 13th day from onset, high spiking fever recurred, and the patient developed rapidly progressive renal function deterioration with hypourocrinia and hematuresis (creatinine level increased to 198 mg/dL). On the 14th day from onset, the patient developed cardiac arrest and received cardiopulmonary resuscitation. Flexible bronchoscopy performed on the 15th day revealed bronchial mucosal erosion and inflammatory small nodule formation (Fig. 4). *Acinetobacter baumannii* was detected in the blood, bronchoalveolar lavage (BAL), and urine cultures. Aspergillus antigen was noted in the blood. Voriconazole and tigecycline were administered according to the pathogen detected. The patient needed MV support for 23 days, and he was febrile for 30 days despite multi-antibiotics and antifungal drugs. After weaning, he was still short of breath and needed supplemental oxygen. The patient had cough with wheezing and dyspnea without oxygen despite discharge.

**Figure 5:**
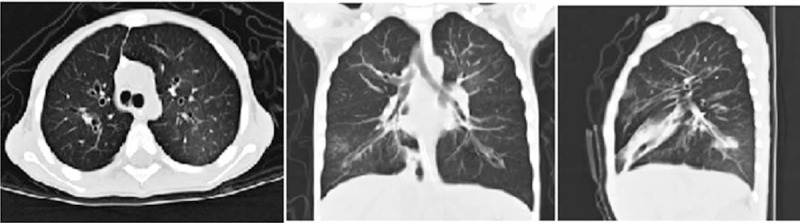
HRCT image showing bilateral bronchi and their branches locally dilated and wall thickened.

HRCT on the 121st day from onset showed bilateral bronchi and their branches were locally dilated and the wall was thickened (Fig. 5). The diagnosis of bronchiectasia was considered. He was prescribed prednisolone 0.5 to 1 mg/kg/day and cyclosporine 25 mg/day for 1 month. However, the child continued to have cough, dyspnea, and wheezing, and his respiratory status progressively worsened. After his first discharge, he was readmitted to the PICU twice because of respiratory distress caused by recurrent severe secondary bacterial infections (*Moraxella catarrhalis* and *Pseudomonas aeruginosa* infections in the lung). He died because of out-of-control severe infections in the lung on the 211th day from the onset of illness during the third time of PICU admission.

### 2.3 Patient 3

**Figure 6:**
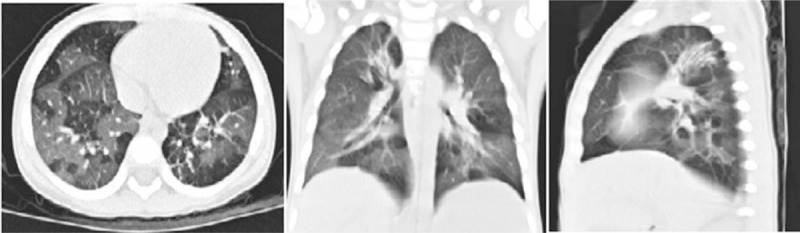
HRCT image showing bilateral mosaic ground-glass patterns with air trapping.

**Figure 7:**
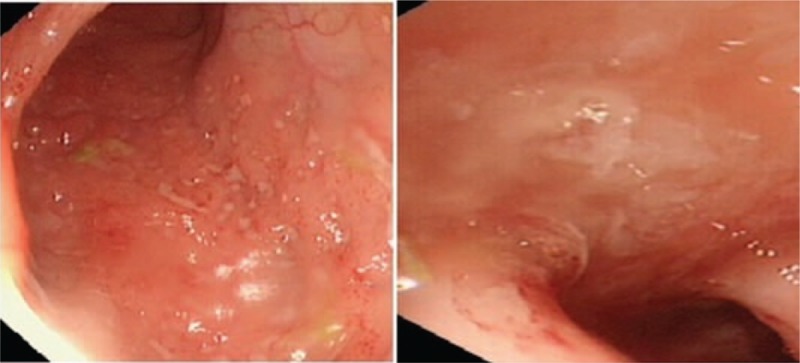
Colonoscopy image revealing congestion, erythema, erosions, superficial ulcerations and mucopurulent exudates affecting the colon and terminal ileum.

A 2-year-old girl was prescribed cefotaxime by a clinic physician because of cough for 1 week. She developed mild fever and rash involving her neck and trunk on the 9th day from the onset of illness. She was admitted to a local hospital and was administered azithromycin for 3 days; however, the symptoms of fever did not improve. She was prescribed cefotaxime again for 5 days. On the 17th day from onset, she developed high spiking fever and a larger area of skin lesions with a widespread vesicular rash that involved approximately 15% of her BAS. A diagnosis of SJS/TEN was considered. She was treated with IVIG 1 g/kg/d for 2 days and methylprednisolone 10 mg/kg/d for 3 days. Despite the temperature returning normal and the gradual healing of skin lesions with desquamation and hyperpigmentation, diarrhea and liver damage (ALT, 312 U/L and AST, 209 U/L) developed. She was transferred to a children hospital in Shanghai on the 25th day from onset. However, diarrhea continued, with a passage of up to 40 loose stools each day with blood and purulent mucus for nearly 2 months. On the 60th day from onset, the patient had cough with wheezing, was short of breath, and was transferred to our hospital. On hospital admission, blood test results were as follows: WBC count, 23.6 × 10^9^/L (NEUT 62%, LYM 20%); platelet count, 409 × 10^9^/L; hemoglobin level, 107 g/L; and CRP level, 3.4 mg/dL. Abnormal serum biochemistry index was observed (ALT, 201 U/L; AST, 198 U/L; and serum albumin, 17 g/L). Routine stool examination showed WBC++++/HPF, RBC +/HPF, and occult blood. *Candida albicans* was detected in the stool culture twice. *Klebsiella pneumoniae* was found in both the blood and sputum cultures. The patient had cow's milk protein allergy. HRCT on the 65th day from onset showed bilateral mosaic ground-glass patterns with air trapping (Fig. 6). Colonoscopy on the 70th day from onset revealed congestion, erythema, erosions, superficial ulcerations, and mucopurulent exudates that affected the colon and also involved the terminal ileum (Fig. 7). The diagnosis of SJS/TEN with obliterans bronchiolitis (BO), hypoproteinemia, liver damage, and enterocolitis were considered. The patient was fed amino acid formula and prescribed multi-antibiotics (tienam, vancomycin, and metronidazole) and antifungal drug (itraconazole) according to the pathogens detected. She was also treated with low doses of methylprednisolone (2 mg/kg/day for 5 days, tapering gradually) to reduce the inflammation. However, the patient still had recurrent fever, and her respiratory and gastrointestinal status progressively worsened. She was transferred to the PICU because of respiratory distress, severe gastrointestinal tract hemorrhage, and septic shock on the 75th day from onset.

On PICU admission, her respiratory rate was 45 breaths/min, blood pressure was 88/45 mmHg, and oxygen saturation was 95% during high flow oxygen. Physical examination revealed skin lesions with desquamation and hyperpigmentation covering approximately 10% of her BSA. She had severe malnutrition and weighed 10 kg (losing 4 kg in 2 months). Bilateral crackles were audible in the chest. Abdominal distension, hepatomegaly, and hypoactive bowel sounds were observed. The distal extremities were cool. Blood test results were as follows: WBC count, 14.2 × 10^9^/L (NEUT 72%, LYM 22%); platelet, 245 × 10^9^/L; hemoglobin, 62 g/L; and CRP, 118.7 mg/dL. Abnormal serum biochemistry index showed an ALT of 78 U/L, AST of 92 U/L, and serum albumin of 26 g/L. Arterial blood gas examination revealed PH of 7.54, hypokalemia of 2.6 mmol/L, PaCO_2_ of 90 mmHg, and PaO_2_ of 90 mmHg during FiO_2_ of 0.6 before MV therapy. Flexible bronchoscopy performed in the PICU on the 76th day from onset revealed obvious bronchial congestion and a large number of white sticky exudates.

Based on the diagnosis of SJS/TEN and respiratory failure, BO, hypoproteinemia, liver damage, enterocolitis, septic shock, and gastrointestinal failure, aggressive support treatments including high-frequency oscillatory ventilation therapy, blood transfusion therapy, and multi-antibiotics and antifungal therapy (meropenem, vancomycin, amikacin, and itraconazole) were administered. However, the severe infection was out of control with extremely high WBC counts (92.6 × 10^9^/L). The patient died 12 days after PICU admission because of multi-organ failure (respiratory and gastrointestinal failure) caused by severe infection in the lung and gastrointestinal tract on the 87th day from onset. Table 1 summarizes these three fatal patients’ characteristics, triggers, laboratory, microbiological, radiological findings, complications, and outcomes.

**Table 1:**
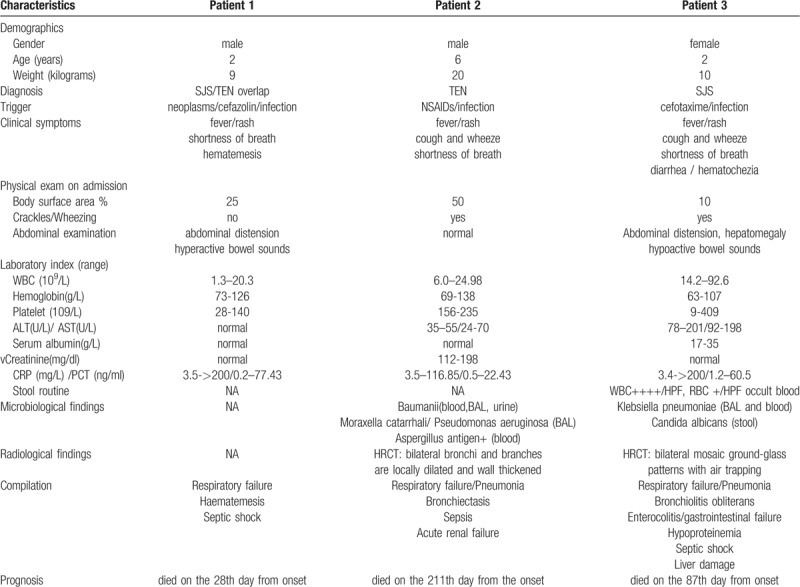
Summary of the characteristics, triggers, laboratory, microbiological, and radiological findings, complications, and outcomes of the three patients. Characteristics of patients with severe SJS and TEN.

## 3 Discussion

SJS/TEN are rare but severe complications with high mortality and are often triggered by drugs such as antiepileptic drugs, antibiotics (aminopenicillins and cephalosporins), chemotherapy, and NSAIDs.^[2,9,10]^ A study showed that antibiotics were the second most frequent cause of fatality in SJS/TEN.^[11]^ Infectious pathogens, including viral and bacterial organisms, have also been associated with this disease spectrum.^[10]^ No trigger is identified in 10% of cases.^[12]^ In our cases, Patients 2 and 3 were triggered by drugs (NSAIDs and cefotaxime) or previous infections. A recent large observational study on the epidemiology of SJS/TEN showed that active neoplasms may be associated with an increased risk of SJS/TEN; however, it is unclear whether the active neoplasms could induce SJS/TEN.^[13]^ The commonest trigger in all identified neoplasm cases of SJS/TEN was antibiotics.^[14]^ For Patient 1 with a diagnosis of skull base neoplasms who developed SJS/TEN, we could not determine whether SJS/TEN was related to previous infections or active neoplasms or was induced by previous use of antibiotics (cefazolin). We should pay attention to active neoplasms that may induce the development of SJS/TEN independently.

Pulmonary complications of SJS/TEN in the chronic phase are BO and bronchiectasia, which are considered severe complications of SJS/TEN^[15]^ among others. BO and bronchiectasia in children are usually caused by severe post-infections in the lung.^[16]^ The causal agents of BO include adenovirus, influenza virus, measles virus, and *Mycoplasma pneumoniae*,^[17]^ while bronchiectasia is often caused by bacteria. However, SJS/TEN with BO and bronchiectasia are not very common.^[15]^ Patients 2 and 3 in our study presented with dyspnea on the 6th and 60th days from the onset of illness, and they both had progressive dyspnea and cough with wheezing in the chronic stage. The diagnosis of BO and bronchiectasia are made based on clinical presentation and HRCT findings. More interestingly, in addition to BO, Patient 2 developed acute renal failure with hematuresis in the acute stage. Severe gastrointestinal involvement (GI) is also a distinctly rare manifestation of SJS/TEN, and the associated mortality is quite high (44%).^[7]^ GI more frequently occurs in the mouth and esophagus and less frequently in the small intestine and colon.^[1,7,18]^ The usual presenting symptoms include bloody and watery diarrhea, abdominal pain, abdominal distension, protein-losing enteropathy, malabsorption syndromes, and hypoalbuminemia.^[7]^ Patient 3 had persistent bloody mucopurulent diarrhea for nearly 2 months with severe malnutrition and hypoproteinemia. She was diagnosed with enterocolitis based on clinical presentation and colonoscopy findings. We speculated that the cause of all these conditions was an immune-mediated reaction that led to severe inflammation occurring in the bronchial, colonic, and urinary mucosa in SJS/TEN patients. The inflammatory changes caused damage and loss of integrity of the mucosal barrier, which increased exposure to bacterial antigens. Therefore, we should be aware that mucosal damage occurs on the skin and within the mucosa of visceral organs, leading to the occurrence of BO, bronchiectasia, enterocolitis, acute renal failure, and severe secondary infections in SJS/TEN.

According to previous literatures, predictors of mortality included renal failure, malignancy, septicemia, any bacterial infection, and epilepsy.^[4]^ A severity-of-illness score for toxic epidermal necrolysis (SCORTEN) is a clinically predictive score used to evaluate the risk of mortality in adult TEN patients, which is based on 7 prognostic factors.^[19]^ There were 3–4 positive indexes of SCORTEN in Patients 1 and 2 (malignancy, surface area involved, heart rate, serum urea). However, this method has not been completely validated in the pediatric population and does not include infection. The pathogenesis of SJS/TEN that incudes multi-organ involvement is not completely clear. It could reflect severe inflammation induced by immune response and infection.^[20]^ Infectious complications were the main cause of morbidity and fatality in acute SJS/TEN.^[21]^ Secondary infections and hospital-acquired infections are thought to be other causes of late deterioration in the chronic stage including MRSA, *P. aeruginosa*, *Stenotrophomonas*, *Acinetobacter*, and *Candida* infections.^[8,22]^ Large skin areas of Patients 1 and 2 became involved, and the children developed severe infections in a very short time in the acute stage. We assumed that septicemia was likely because of the impaired mucosal barrier from denuded skin. *Acinetobacter baumannii*, *M. catarrhalis*, *P. aeruginosa*, and *K. pneumoniae* were detected in BAL cultures of both Patients 2 and 3, and *C. albicans* was detected in the stool culture of Patient 3 in the chronic stage. All these 3 patients died because of septic shock caused by out-of-control severe infections. Given these conditions, prospective studies of SJS/TEN with severe complication are required to establish a clinically predictive score that includes severe infection for pediatric patients to evaluate the risk of mortality in children.

There is no consensus regarding the optimal treatment for SJS/TEN.^[2]^ Although systemic corticosteroids are frequently used, it is controversial, and large-scale studies are lacking.^[1,2]^ Corticosteroids only mitigate mucosa edema and improve the symptoms temporarily.^[23]^ According to a recent systematic review and meta-analysis, no benefit could be seen in patients treated with high-dose IVIG; however, in terms of mortality, the early use of IVIG was shown to be effective.^[24]^ Immunosuppressants such as cyclosporine are also used for treating SJS with BO, although the effect is uncertain.^[25]^ Following the diagnosis of SJS/TEN, all three patients received systemic corticosteroids and IVIG in the acute stage, and Patients 2 and 3 received oral prednisolone or low doses of methylprednisolone in the chronic stage. Patient 2 also received cyclosporine therapy in the chronic stage. However, these treatments did not seem to be effective. The patients’ status gradually worsened. Moreover, the effects of corticosteroids, IVIG, and immunosuppressants were uncertain. Large-scale studies are needed to evaluate their effects on SJS/TEN.

## 4 Conclusion

In conclusion, SJS/TEN are rare, and their severe complications are significantly associated with high mortality. We should be aware that mucosal damage occurs on the skin and within the mucosa of visceral organs, leading to the occurrence of bronchiectasia, BO, enterocolitis, acute renal failure, and severe secondary infections. Prospective studies are required to evaluate the effects of corticosteroids, IVIG, and immunosuppressants to SJS/TEN and establish a clinically predictive score that includes severe infection for pediatric patients to evaluate the risk of mortality in children in order to improve poor outcomes.

## Author contributions

**Conceptualization:** Gen Lu.

**Data curation:** Li Huang, Huifeng Fan.

**Formal analysis:** Huan Chen.

**Methodology:** Diyuan Yang.

**Resources:** Huan Chen, Li Huang, Dongwei Zhang.

**Software:** Diyuan Yang.

**Supervision:** Dongwei Zhang.

**Visualization:** Huifeng Fan.

**Writing – original draft:** Tingting Shi.

**Writing – review & editing:** Tingting Shi, Gen Lu.
